# Host factors in parainfluenza virus replication: from entry to innate immunity evasion

**DOI:** 10.3389/fimmu.2026.1823377

**Published:** 2026-05-28

**Authors:** Xueqin Lin, Yun Zhu, Ling Jing, Xia Xiao, Xiaobo Lei, Zhengde Xie

**Affiliations:** 1 Laboratory of Infection and Virology, Beijing Pediatric Research Institute, Beijing Children’s Hospital, Capital Medical University, National Center for Children’s Health, Beijing, China; 2 Beijing Key Laboratory of Core Technologies for the Prevention and Treatment of Emerging Infectious Diseases in Children, Beijing, China; 3 Key Laboratory of Major Diseases in Children, Ministry of Education, National Clinical Research Center for Respiratory Diseases, Beijing, China; 4 Research Unit of Critical Infection in Children, Chinese Academy of Medical Sciences, Beijing, China; 5 NHC Key Laboratory of System Biology of Pathogens and Christophe Merieux Laboratory, National Institute of Pathogen Biology, Chinese Academy of Medical Sciences and Peking Union Medical College, Beijing, China

**Keywords:** host factors, host-directed therapy, human parainfluenza virus, innate immunity, viral life cycle, virus-host interactions

## Abstract

Human parainfluenza viruses (HPIVs)are significant pathogens responsible for acute respiratory infections in infants, the elderly, and immunocompromised individuals. Among children under five, HPIVs are the second most common viral cause of acute lower respiratory tract infections (ALRIs), surpassed only by respiratory syncytial virus (RSV). Despite their significant health impact, there are currently no licensed vaccines or specific antiviral treatments for HPIV infection. Recent advances in functional genomics, including CRISPR-Cas9 screening, cDNA library screening, and multi-omics approaches, have enabled the systematic identification of host factors essential at different stages of HPIVs infections. This review aims to systematically summarize the latest evidence on host factors that influence viral entry, replication, assembly, and release, as well as those involved in evasion of innate immunity. Importantly, we contextualize the mechanisms of these host factors within the broader network of virus-host interactions. By integrating these insights, we aim to provide a strong mechanistic foundation for understanding HPIV pathogenesis to accelerate the development of innovative host-directed therapies(HDTs).

## 1 Introduction

HPIVs are significant pathogens responsible for ALRIs in children under five years of age worldwide, with their detection rates ranking second only to RSV. Among the four HPIV types (HPIV1-4), HPIV1 and HPIV3 are the primary causative agents of bronchiolitis and pneumonia. Globally, HPIVs contribute substantially to the burden of ALRIs, accounting for approximately 13% of all ALRI cases, 4-14% of ALRI-related hospitalizations, and 4% of ALRI-associated mortality among children under five years old (1). Currently, clinical management entirely relies on supportive therapies due to the absence of approved vaccines or specific antivirals. The rapid emergence of drug resistance, driven by the high mutation rates typical of RNA viruses, severely complicates the development of antivirals. Consequently, targeting highly conserved host factors, cellular proteins essential for the viral life cycle, presents a compelling strategy to develop broad-spectrum antivirals with a higher genetic barrier to resistance (2).

**Figure 1:**
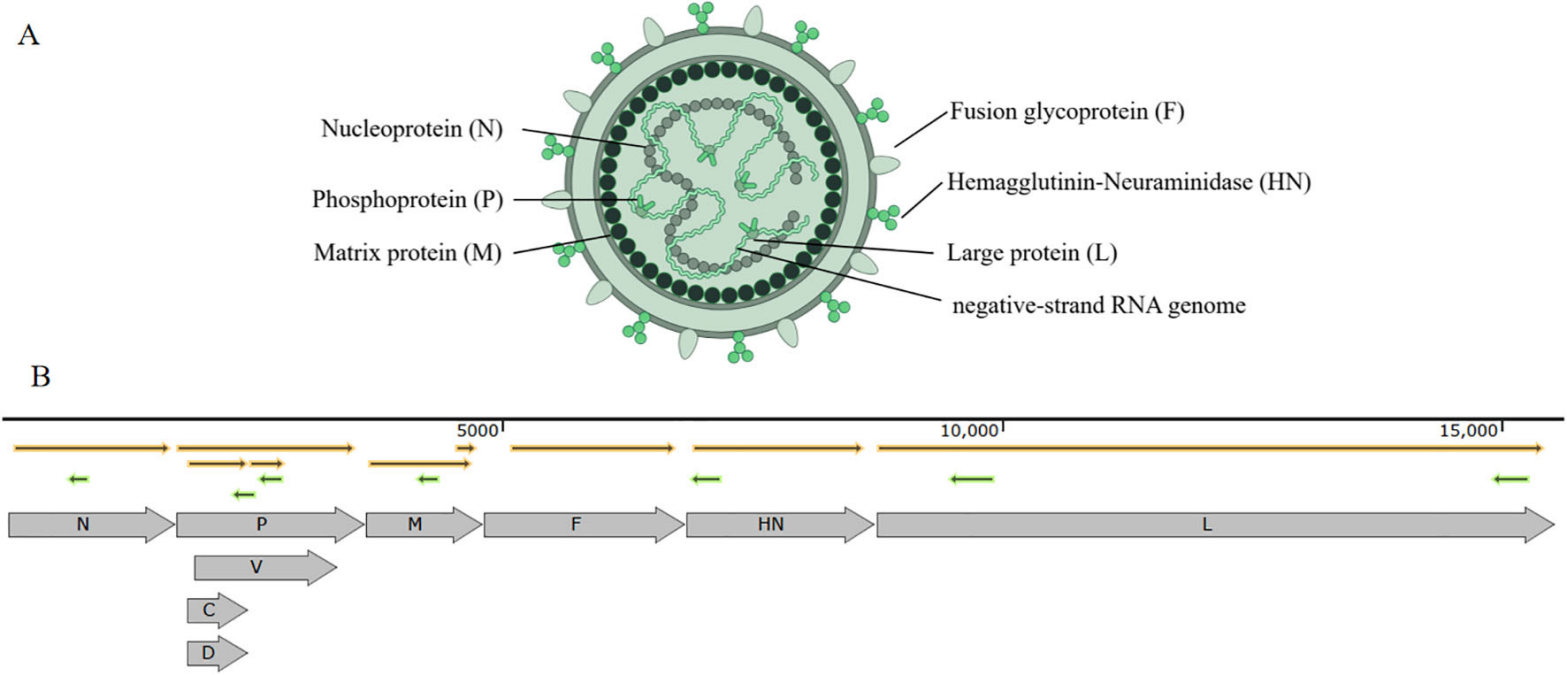
Schematic of HPIV structure and genome. **(A)** Structural overview of HPIV. The viral envelope, derived from the host cell membrane, is studded with the fusion (F) and hemagglutinin-neuraminidase (HN) glycoproteins. Underlying the envelope, the matrix (M) protein forms a shell that encloses the helical ribonucleocapsid core, which consists of the viral RNA bound to the nucleocapsid (N) protein, the phosphoprotein (P), and the large polymerase subunit (L). The accessory proteins C, V, and D varies among HPIV types. (By Figdraw) **(B)** Genome of HPIV. The schematic illustrates the single-stranded, negative-sense RNA genome. The P gene, in particular, can give rise to multiple non-structural proteins (including C, V, and D) through mechanisms such as RNA editing and alternative translational initiation.

HPIVs, belonging to the family *Paramyxoviridae*, are enveloped, single-stranded negative-sense RNA viruses. They are primarily classified into four types (HPIV1–4). Notably, HPIV1 and HPIV3 are categorized within the genus Respirovirus, whereas HPIV2 and HPIV4 (including HPIV4A and HPIV4B) belong to the genus Rubulavirus. The viral genome, approximately 14.9–17.3 kb in length, encodes six major structural proteins in the conserved order 3’-N-P-M-F-HN-L-5’: nucleoprotein (N), phosphoprotein (P), matrix protein (M), fusion protein (F), hemagglutinin-neuraminidase (HN), and the large RNA-dependent RNA polymerase (L) (**Figure 1**). Among these, the envelope glycoproteins HN and F mediate receptor recognition and membrane fusion, respectively. The genomic RNA is tightly encapsidated by N to form the ribonucleoprotein (RNP) complex, which together with the P and L proteins, constitutes the active RNA-dependent RNA polymerase complex. The M protein orchestrates assembly by bridging the RNP and the cytoplasmic tails of the envelope glycoproteins. Furthermore, via RNA editing and overlapping or alternative open reading frames, the P gene encodes three non-structural accessory proteins, including C, V, and D proteins. These non-structural proteins act as critical virulence factors that modulate host innate immunity, specifically the interferon response, facilitating viral replication (3) (**Figure 1**). Throughout their life cycles, HPIVs engage a vast network of host factors through both structural and non-structural proteins, enabling efficient infection, replication, and transmission within the human respiratory tract (**Table 1**).

**Table 1:** Key host factors of the HPIV life cycle and functions.

Replication cycle		Host factor	Targeted viral protein	Virus type	Functional module	Mechanism	Reference
Entry		SA	HN	HPIV1, 3	Receptor binding	Serves as the initial attachment receptor, mediating viral binding to the host cell surface	(4, 5)
		Nucleolin	F	HPIV3	Receptor binding	Acts as a co-receptor, interacting with the F protein to facilitate viral entry	(6, 7)
		TMPRSS2 TMPRSS13	F	HPIV3	Proteolytic activation	Triggers membrane fusion by cleaving the F protein; a critical activation step for viral entry	(8–13)
Transcription & Translation	Promotion	GTP	L	HPIV2	Molecular mimicry	Viral L protein directly mediates viral mRNA capping by mimicking the GTP-binding domain of host capping enzymes	(14)
		α-tubulin	NP	HPIV3	Cytoskeletal regulation	Viral NP complex recognizes acetylated microtubules and enters the microtubule lumen to promote inclusion body fusion and RNA synthesis	(15)
		PI4KB	P	HPIV3	Phase separation & replication compartment organization	Recruited by the P protein to inclusion bodies formed via phase separation; generates a PI4P-enriched microenvironment that stabilizes condensates and enhances RNA synthesis	(16)
	Inbibition	VIM	NP	HPIV3	Cytoskeletal regulation	Opposes the above process by promoting α-TAT1 degradation and reducing microtubule acetylation	(17)
		IDO1	/	HPIV3	Metabolic reprogramming	Depletes tryptophan, restricting viral protein synthesis through amino acid metabolic regulation	(18, 19)
		PKR	C	HPIV3	Translation control	Activated by viral dsRNA; phosphorylates eIF2α to block cap-dependent translation initiation	(19, 20)
		MxA	L	HPIV3	Effector functions	Binds to viral RNP after primary transcription of HPIV3, inhibiting early viral transcription	(21, 22)
		MxB	RNP	HPIV1	Effector functions	Interacts with the HPIV1 RNP complex	(23)
Assembly & Release	Promotion	RhoA	V	HPIV2	RhoA signaling	Activates the RhoA signaling pathway, promoting F-actin polymerization to provide structural support for viral assembly	(24, 25)
		PFN2	V	HPIV2	RhoA signaling	Cooperates in promoting actin polymerization by activating the RhoA signaling pathway	(26)
		Rab11a	RNP	HPIV3	Vesicular transport	Mediates vesicle fusion upon GTP binding, participating in intracellular transport of viral RNP	(27, 28)
		Rab27a	HN/F	HPIV2	Vesicular transport	Regulates vesicle anchoring and release, mediating transport of viral glycoproteins to budding sites	(29)
		Alix	V	HPIV2	ESCRT-mediated budding	Recruits the ESCRT complex to mediate membrane scission and viral particle release from the cell membrane	(30)
		Cavin3	V	HPIV2	Lipid raft organization	Enhances formation of lipid raft microdomains, providing a cholesterol- and sphingolipid-enriched platform for viral budding	(31)
	Inhibition	CLDN1	/	HPIV2	Physical restriction	Forms a physical barrier at tight junctions, restricting viral spread	(32, 33)
		Graf1	P/V	HPIV2	RhoA signaling	Inactivates RhoA via its GAP activity, antagonizing actin polymerization and inhibiting viral assembly	(25, 34)
		SNAP29	V	HPIV3	Autophagy regulation	Participates in SNARE complex formation, driving autophagosome-lysosome fusion to complete autophagic flux	(35–37)
		BST-2	V	HPIV2	Physical restriction	Tethers newly formed viral particles to the infected cell surface, preventing their release	(38–41)
Antiviral Innate Immunity		TRAF6	V	HPIV2	Signal transduction	Mediates K63-linked polyubiquitination of IRF7, activating the TLR7/9 signaling pathway	(42)
		STAT1	C	HPIV1, 3	Transcriptional regulation	Translocates to the nucleus upon phosphorylation to initiate transcription of interferon-stimulated genes.	(43–45)
		STAT2	V	HPIV2	Transcriptional regulation	Forms the ISGF3 complex with IRF9 to mediate type I/III interferon signaling	(46, 47)
		MDA5	V	HPIV2	Pattern recognition	Recognizes viral RNA and activates RLR signaling pathways to initiate interferon production	(48, 49)
		TBK1	V	HPIV2	Signal transduction	Phosphorylates IRF3/IRF7 and activates NF-κB; a central kinase for interferon induction	(50)
		TDRD7	/	HPIV3	Autophagy regulation	Induced by interferon; inhibits AMPK phosphorylation to block virus-induced autophagy	(51, 52)

Recent technological breakthroughs, including CRISPR/Cas9-based functional screening, high-throughput plasmid cDNA library screening, and mass spectrometry-based proteomics, have accelerated the discovery of HPIV host factors involved in the entire HPIV life cycle. This review aims to systematically summarize these findings, focusing on the host factors required for viral binding, entry, replication, assembly, and release, as well as those involved in innate immune evasion and the development of novel antiviral interventions.

## 2 Host factors involved in HPIV life cycle

### 2.1 HPIVs entry

**Figure 2:**
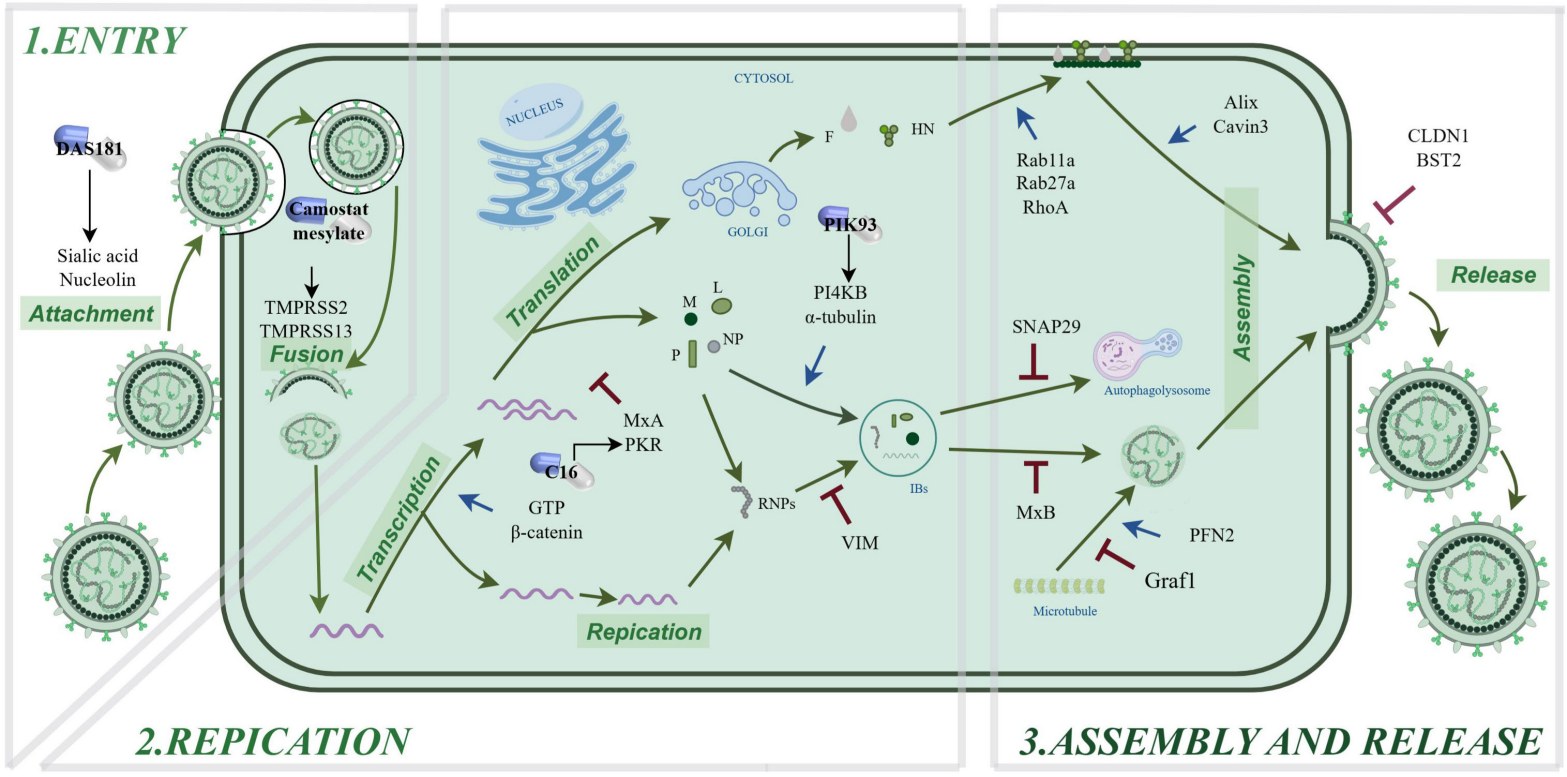
Schematic overview of host factors involved in the HPIV life cycle. This figure illustrates the key host factors that interact with HPIV at different stages of the viral life cycle, including entry, replication, assembly, and release. Host factors are categorized according to their functional roles, with arrows indicating the direction of the viral life cycle. The blue arrow indicates promotion of viral replication, whereas the flat-head arrow indicates antiviral restriction functions. Abbreviations are defined in the main text. represents related antivirals/inhibitors. (Thanks for Figdraw).

HPIV entry is a pH-independent process initiated by viral attachment to host surface receptors, which is a critical step that strictly determines viral tropism and pathogenesis. Multiple key host factors involved in HPIV entry have been identified. Among these, sialic acid serves as the primary functional receptor, while accessory factors like nucleolin and host proteases cooperate to facilitate viral invasion (**Figure 2**).

#### 2.1.1 Sialic acid

Sialic acid residues, a widely distributed neuraminic acid derivative, serve as the primary receptor for HPIV attachment. Typically conjugated to the termini of cell surface glycoproteins or glycolipids via α-2,3 or α-2,6 glycosidic linkages, SA plays a critical role in cell adhesion and immune modulation. The viral HN protein binds to SA on the cell surface, anchoring the virion to the plasma membrane. This binding event induces a conformational change in the adjacent F protein, triggering viral–host membrane fusion and facilitating viral entry. Receptor linkage specificity strongly dictates viral tropism. The HN protein of HPIV3 exhibits broad binding capacity, recognizing both α-2,3- and α-2,6-linked sialic acids (4), which correlates with its wide spectrum of tissue tropism. In contrast, the HPIV1 HN possesses a distinct structural configuration with dual binding sites, limiting its specificity to α-2,3-linked (5) and certain α-2,8-linked sialic acids (53). This differential receptor specificity underlies the significant variations in tissue tropism and pathogenesis between these two types. Consequently, host-directed therapies that enzymatically remove sialic acid receptors, such as the recombinant sialidase DAS181, represent a highly promising antiviral strategy. DAS181 is currently under evaluation in Phase III clinical trials for HPIV in ALRIs immunocompromised patients (54, 55).

#### 2.1.2 Nucleolin

Nucleolin is a multifunctional RNA-binding protein primarily localized to the nucleolus, though it dynamically shuttles to the cytoplasm and plasma membrane (56). While it acts as a receptor for various physiological ligands, cell surface nucleolin is critical for HPIV3 entry. HPIV3 infection can proceed via nucleolin even in the absence of canonical sialic acid, indicating its capacity to potentially function as an independent receptor (57, 58). Mechanistically, the F protein of HPIV3 specifically interacts with nucleolin enriched at the apical plasma membrane. This interaction acts synergistically with sialic acid to significantly enhance the efficiency of viral entry and promote internalization (6, 7), identifying that cell surface nucleolin serves as a crucial receptor accessory protein in HPIV3 entry.

#### 2.1.3 Serine proteases

Serine proteases, which utilize serine residues at their catalytic center, are a class of enzymes involved in various physiological processes such as blood coagulation, immune regulation, and tissue remodeling. For HPIV, host serine proteases constitute an essential dependency host factor required for the proteolytic activation of the viral F protein, a step necessary for the virus to establish infection The F protein is initially synthesized as an inactive precursor (F0) (59). Fusion competence is achieved only following cleavage by host serine proteases at a specific site, generating the disulfide-linked F1 and F2 subunits (60). This cleavage is an absolute prerequisite for the protein to mediate fusion between the viral and cellular membranes, permitting RNP entry into the cytoplasm (61, 62). Transmembrane serine proteases 2 and 13 (TMPRSS2 and TMPRSS13) from the type II transmembrane serine protease family are highly expressed in the human respiratory epithelium (8, 9). These homologous proteases have been demonstrated to directly cleave and activate the HPIV3 F protein, significantly driving viral entry (10–13). Given the critical role of TMPRSS2 in respiratory virus invasion, its inhibitors (e.g., camostat mesylate), which have been extensively studied in COVID-19, warrant further exploration for repurposing against HPIVs (63, 64).

### 2.2 Host factors related to viral gene transcription and translation

Upon completing entry, HPIVs efficiently hijack the host transcriptional and translational machinery to enable viral gene expression. This process is orchestrated through elegant strategies such as molecular mimicry, cytoskeletal coordination, and the spatial remodeling of the membrane microenvironment, allowing the virus to systematically subvert or counteract specific host factors. At the cytoskeletal level, α-tubulin acetylation facilitates the transport and spatial organization of viral RNPs, a process counteracted by vimentin (VIM). Metabolically, phosphatidylinositol 4-kinase β (PI4KB) alters local lipid compositions to provide a distinct lipid microenvironment for viral RNA synthesis. At the translational level, the protein kinase R (PKR)-phosphorylates eukaryotic initiation factor 2α(eIF2α) axis exerts a critical restrictive function by shutting down cap-dependent translation, while the virus mimics host capping using intracellular guanosine triphosphate (GTP). Additionally, indoleamine 2,3-dioxygenase 1 (IDO1) restricts viral protein synthesis by rewiring amino acid metabolism. Collectively, these factors form an intricate tug-of-war during HPIV replication (**Figure 2**).

#### 2.2.1 Pro-viral host factors

##### 2.2.1.1 Guanosine triphosphate

GTP is a purine nucleotide that functions both as an energy currency and as a critical donor for the 5’ cap modification of host mRNA, a process essential for mRNA stability, nuclear export, and translation initiation. During HPIV infection, the virus exploits this host capping machinery through molecular mimicry. Specifically, a highly conserved domain near the C-terminus of the HPIV2 L protein shares striking structural similarity with the GTP-binding region of host capping enzymes. This enables L protein of HPIV to directly sequester intracellular GTP and autonomously catalyze the capping of viral mRNA, expertly bypassing the host’s capping enzyme complex (14).

##### 2.2.1.2 α-tubulin

α-tubulin is one of the fundamental subunits of microtubules. As a crucial element of the cytoskeleton, it interacts synergistically with intermediate filaments and microfilaments, thereby modulating multiple stages of the viral life cycle (65). Notably, the acetylation of lysine 40 (K40) on the luminal surface of microtubules is critical for maintaining their structural stability and modulating their functions (66). It has been demonstrated that the HPIV3 N-P complex specifically recognizes this acetylated K40 site on α-tubulin. Following this recognition, the complex enters the microtubule lumen, thereby facilitating the fusion of viral inclusion bodies (IBs) and enhancing viral RNA synthesis (15).

##### 2.2.1.3 Phosphatidylinositol 4-kinase β

PI4KB, a member of the phosphatidylinositol kinase family, catalyzes the phosphorylation of phosphatidylinositol (PI) to generate phosphatidylinositol 4-phosphate (PI4P), which is a lipid crucial for intracellular membrane transport. During HPIV3 infection, the viral P protein actively recruits PI4KB to the IBs (16). Notably, IBs are dynamic biomolecular condensates formed via liquid-liquid phase separation, a process that assembles viral RNA, polymerases, and host cofactors into membraneless replication factories. This recruitment of PI4P drives its localized accumulation around these condensates, thereby establishing a specialized lipid microenvironment. This microenvironment physically stabilizes the phase-separated IBs and insulates the viral RNA synthesis machinery from cytosolic sensors (16). Because of its critical role, PI4KB may be a potential antiviral target. While its specific inhibitor, PIK93, demonstrates potent anti-HPIV activity *in vitro*, PI4KB’s involvement in essential cellular trafficking pathways means that its broad-spectrum cytotoxicity and *in vivo* efficacy require rigorous validation (67).

#### 2.2.2 Antiviral restriction host factors

##### 2.2.2.1 Vimentin

VIM, a type III intermediate filament protein, constitutes an essential component of the cytoskeleton and is involved in maintaining cellular structure as well as multiple. The specific binding between VIM and the N-P complex of HPIV3 occurs via amino acid residues 61–80 within its head domain, leading to inhibition of viral gene transcription. Short peptides designed to target this region have demonstrated potent antiviral activity (17). Furthermore, VIM also suppresses IBs fusion and maturation by promoting the degradation of α-TAT1 and reducing microtubule protein acetylation levels.

##### 2.2.2.2 Indoleamine 2,3-dioxygenase 1

IDO1 is the rate-limiting enzyme in the kynurenine pathway of tryptophan metabolism. By depleting tryptophan in the local microenvironment, IDO1 triggers a stress response that arrests protein synthesis and suppresses viral replication (18). In human epithelial cells, IDO1 upregulation potently suppresses HPIV3 replication, and this effect is reversed by exogenous tryptophan supplementation (19). Beyond this mechanism, HPIV infection has been increasingly recognized to induce broader metabolic reprogramming of host cells, encompassing alterations in glycolysis, glutaminolysis, and lipid metabolism. IDO1-mediated tryptophan catabolism represents one node within this larger metabolic rewiring, highlighting how viruses and hosts compete for metabolic resources (68).

##### 2.2.2.3 Protein kinase R

PKR is a double-stranded RNA (dsRNA)-activated kinase that serves as a sentinel against viral infection. Upon activation by viral dsRNA, PKR phosphorylates eIF2α, which in turn sequesters the guanine nucleotide exchange factor eIF2B, leading to suppression of translation initiation. Since most viruses, including HPIV3, rely on host cap-dependent translation for viral protein synthesis, PKR activation imposes a potent antiviral barrier (19, 20). Based on the antiviral function of PKR, C16 (a PKR activator) can inhibit viral replication by activating PKR, although their pro-apoptotic risk requires careful evaluation. In contrast, 2-AP (a PKR inhibitor) is primarily used as an experimental tool for mechanistic studies of this pathway (69).

##### 2.2.2.4 Myxovirus resistance protein A and B

The Mx proteins, including MxA and MxB, are dynamin-like GTPases induced by type I and III interferons, serving as robust effectors of the antiviral defense system. MxB localizes to the nuclear membrane and the cytoplasm (70), while MxA accumulates in the cytoplasm (21, 71–73). Experimentally, MxB has been shown to interact with the RNP complex of HPIV1, contributing to a role in disrupting viral assembly (23). In contrast, MxA inhibits HPIV3 replication at an early stage, specifically blocking primary transcription (21, 22). These distinct spatial and temporal mechanisms highlight the sophisiticated, multi-tiered nature of host innate immunity against distinct HPIV types.

### 2.3 Host factors associated with HPIVs assembly and release

The efficient assembly and release of viral particles represent the late stage of the HPIV life cycle, which is supported by host cellular machinery. The cytoskeleton provides a structural framework. Viruses utilize the host endosomal system to achieve the targeted transportation and processing of their envelope glycoproteins. Additionally, the virus directly recruits host membrane scission machinery, including the endosomal sorting complex required for transport (ESCRT), to mediate the final stage of viral egress.

#### 2.3.1 Host factors that promote viral assembly and release

##### 2.3.1.1 Ras homolog family member A

RhoA is a key member of the Rho GTPase family, regulating cytoskeletal dynamics, cell morphology, and motility by cycling between an active GTP-bound state and an inactive GDP-bound conformation. This regulation can indirectly influence membrane fusion events between viruses and host cells (24). The HPIV2 V protein specifically binds inactive RhoA, forcefully activating the RhoA signaling pathway and promoting F-actin polymerization. Consistent with this mechanism, treatment with RhoA pathway inhibitors suppresses HPIV2-induced F-actin formation and reduces viral titers. Viral protein expression remains unchanged under these conditions, indicating that RhoA signaling specifically functions during the assembly and release stages of the viral life cycle (25). Given the critical role of RhoA in viral assembly, Y-27632, a ROCK inhibitor targeting the RhoA downstream effector Rho-associated coiled-coil containing protein kinase (ROCK), effectively blocks F-actin polymerization and has been used to validate the antiviral potential of this pathway *in vitro (*74).

##### 2.3.1.2 Profilin 2

PFN2, a member of the profilin protein family, is an actin-binding protein that promotes the polymerization of G-actin into F-actin. The process is critical for viral structure maintenance and replication (26). The HPIV2 V protein directly binds PFN2, displacing it from G-actin to drive downstream RhoA -mediated F-actin formation. Conversely, PFN2 does not interact with HPIV2 transcriptional or translational components. Knockdown of PFN2 impaired F-actin formation and reduced viral titers without affecting viral protein expression, suggesting that PFN2 functions at a post-translational stage of the viral life cycle (26).

##### 2.3.1.3 Rab GTPases

Rab11a and Rab27a, members of the Rab GTPase family, regulate cellular vesicle transportation (75, 76). Rab11a mediates vesicle fusion via microtubules or actin-dependent mechanisms, thereby modulating intercompartmental protein transport (27, 77, 78). The RNP of HPIV3 can specifically recognize the GTP-bound form of Rab11a. Through the actin-mediated endosomal transport system, RNPs are targeted to the apical membrane to promote progeny virus assembly (28). Meanwhile, Rab27a facilitates the transport of HN and F proteins of HPIV2 to the plasma membrane by regulating vesicle anchoring and release (29).

##### 2.3.1.4 ALG-2-interacting protein X

Alix, a calcium-binding protein, is widely involved in apoptosis and endosomal trafficking. It can recruit the ESCRT complex to mediate endosomal transport and multivesicular body (MVB) formation (79). This process involves the ESCRT-dependent inward budding of the endosomal membrane to generate intraluminal vesicles. This mechanism is highly similar to viral budding (80). In the context of paramyxovirus replication, the ESCRT has been established as essential for efficient viral budding. Correspondingly, Alix has been demonstrated to positively regulate HPIV2 propagation. Its knockdown inhibits viral release, while its overexpression rescues the replication defect in budding-deficient mutants (30).

##### 2.3.1.5 Caveolae-associated protein 3

Cavin3, a member of the Cavin protein family, is primarily involved in the formation and stabilization of caveolae (81). Caveolae are specialized plasma membrane microdomains enriched in cholesterol and sphingolipids, which function as a distinct subtype of lipid rafts. These structures play critical roles in membrane trafficking, signal transduction, lipid metabolism, and the replication cycles of various viruses (82). Notably, the HPIV2 V protein binds to Cavin3, thereby masking its ubiquitination sites. This interaction inhibits Cavin3 degradation and enhances its stability. By stabilizing Cavin3, this interaction promotes membrane remodeling of caveolae and enhances the formation of HPIV2 budding sites within lipid raft microdomains (31).

#### 2.3.2 Host factors that inhibit viral assembly and release

##### 2.3.2.1 Tight junction protein 1 (Claudin-1, CLDN1)

CLDN1, a core component of the tight junction (TJ) complex, is a critical four-pass transmembrane protein that maintains the barrier function and regulates the paracellular permeability of epithelial layers. In the human respiratory epithelium, CLDN1 expression is physiologically relevant to viral infection. Its knockdown enhances the apical egress of HPIV2, whereas its overexpression significantly restricts it (32). Furthermore, TNFα-mediated upregulation of CLDN1 may further potentiate antiviral immunity to HPIV2 (33).

##### 2.3.2.2 GTPase regulator associated with focal adhesion protein 1

Graf1, also known as OPHN1L or ARHGAP26, is a cytoplasmic signaling scaffold protein that functions as a GTPase-activating protein (GAP), catalyzing the inactivation of RhoA (83). During HPIV2 infection, Graf1 inactivates RhoA to suppress F-actin polymerization via its GTPase activity. However, HPIV2 effectively antagonizes this host defense mechanism through directly interacting with Graf1 via viral P and V proteins, which counteract the inactivation of RhoA (25, 34).

##### 2.3.2.3 Synaptosome-associated protein 29

SNAP29 is a member of the N-ethylmaleimide-sensitive factor attachment protein receptor (SNARE) family. As a key adaptor protein that drives the fusion of autophagosomes and lysosomes by bridging syntaxin 17 (STX17) and vacuole-associated membrane protein 8 (VAMP8) (35). The accumulation of autophagosomes has been shown to promote the release of viral particles (36). The HPIV3 P protein binds to SNAP29 via its N-terminal 100-amino acid domain. This binding competitively disrupts the STX17–SNAP29–VAMP8 complex, thereby impairing autophagosome–lysosome fusion. As a result, autophagosomes accumulate and are subsequently redirected by the virus to facilitate the release of extracellular virions (37).

##### 2.3.2.4 Bone marrow stromal cell antigen 2

BST-2, also known as Tetherin/CD317/HM1.24, is an interferon-induced type II transmembrane protein that exhibits broad-spectrum inhibitory activity against a variety of enveloped viruses. By utilizing its terminal anchor domains to tether the viral envelope to the cell membrane, BST-2 retains newly assembled viral particles at the cell surface, thereby preventing their release (38). Furthermore, these tethered virions can be internalized into endosomal compartments, further restricting viral spread (39). To counter this, the HPIV2 V protein specifically binds the glycosylphosphatidylinositol anchor region of BST-2 and promotes its cell-sruface downregulation, successfully releasing progeny virons (40, 41).

## 3 Host factors involved in antiviral innate immunity evasion

HPIV employs highly sophisticated strategies to evade innate immune detection, deploying its non-structural proteins to dismantle type I interferon (IFN) induction and downstream signaling. Moreover, HPIV actively subverts cellular survival pathways, such as autophagy and apoptosis, to maintain a permissive replicative niche.

### 3.1 Tumor necrosis factor receptor-associated factor 6

TRAF6 is a critical intracellular signaling adaptor with E3 ubiquitin ligase activity (84). It transduces signals from Toll-like receptors (TLRs) to activate NF-κB and drive the production of inflammatory cytokines. The pathophysiology of infectious diseases, tumors, and autoimmune diseases is all closely linked to dysfunction of this pathway (85). By specifically binding to TRAF6, the HPIV2 V protein physically blocks the TRAF6-mediated K63-linked polyubiquitination of IRF7. This effectively severs the TLR7/9 signaling cascade, drastically reducing the production of type I interferons (42, 86).

### 3.2 Signal transducer and activator of transcription 1

STAT1 is a master cytoplasmic transcription factor essential for the antiviral state. Upon activation by type I, II, and III interferons, STAT1 is phosphorylated, dimerized, and translocated to the nucleus to drive the massive expression of interferon-stimulated genes (ISGs) (87, 88). To antagonize this vital host defense, the C proteins of HPIV1 and HPIV3 bind to STAT1 steadily, preventing its phosphorylation and subsequent nuclear translocation. This systemic blockade ensures viral persistence by suppressing interferon signaling (43–45).

### 3.3 Signal transducer and activator of transcription 2

STAT2 is another core component of the type I and type III interferon response (89–91). Unlike HPIV1 and HPIV3, which block STAT translocation, the HPIV2 V protein targets STAT2 to promote the ubiquitination and subsequent proteasomal degradation by its E3 ubiquitin ligase activity. The IFN-α/β signaling pathway is effectively blocked by this targeted degradation, which makes it easier for the virus to evade host innate immunity (46, 47).

### 3.4 Melanoma differentiation-associated protein 5

MDA5 is a crucial cytosolic sensor that detects viral dsRNA using its C-terminal helicase domain, subsequently triggering the RIG-I-like receptor (RLR) cascade to produce type I interferon (92). The HPIV2 V protein interacts directly with MDA5, to thwart this host defense. This interaction prevents MDA5 from detecting the viral RNA, blocking the initiation of the RLR signaling pathway and enabling the virus to evade innate immune detection (48). Experimentally, exogenous expression of MDA5 can significantly activate the IFN-β promoter, which is swiftly subverted in the presence of the HPIV2 V protein (49, 93).

### 3.5 TANK-binding kinase 1

TBK1, a serine/threonine kinase belonging to the inhibitor of kappa B kinase (IKK) family, plays a central role in innate immunity by bridging sensor activation with the IRF3/IRF7 phosphorylation and NF-κB induction (94). The HPIV2 V protein functions as an alternative substrate for IKKe/TBK1, competitively inhibiting IRF3 activation and interferon induction. While this disrupts host antiviral signaling, the phosphorylation of the viral V protein itself subsequently targets it for degradation, thus creating a regulatory feedback loop that attenuates virus-induced immune suppression (50). TBK1, as a central kinase in interferon signaling, inhibiting TBK1 may attenuate host antiviral immunity. Therefore, directly targeting TBK1 for antiviral therapy warrants great caution (95).

### 3.6 Tudor domain containing 7

TDRD7 is a multifunctional ISG involved in mRNA metabolism, translation regulation, and stress responses (96). Following initial HPIV3 infection, the key autophagy-initiating kinase AMPK is activated to induce cellular autophagy to support viral replication. However, once the innate immune system catches up, it substantially upregulates TDRD7 (51). TDRD7 acts as a potent antiviral brake by directly inhibiting AMPK phosphorylation and kinase activity. This suppresses the virus-induced autophagy, ultimately inhibiting HPIV3 replication (52).

## 4 Balancing feasibility, toxicity and clinical translation in host targeted therapies of HPIV

Targeting host factors offers a new paradigm for anti-HPIV therapy, but its development requires a careful balance among feasibility, toxicity, and specificity.

Feasibility hinges on identifying host proteins that are indispensable for viral replication yet non-essential for normal host cell survival and function. Among current candidates, α-tubulin (a putative host factor with infection-dependent essentiality) exhibits promising feasibility, as virus-infected cells are much more dependent on it than resting cells, potentially providing a wider therapeutic window (15, 66). Another target worth attention is TMPRSS2, whose expression is relatively restricted to lung epithelial cells, making it safer; nevertheless, long−term inhibition may still interfere with normal physiological functions such as hormonal regulation and epithelial barrier maintenance (11). In contrast, although PI4KB effectively inhibits HPIV3 RNA synthesis, this kinase is also involved in multiple intracellular membrane trafficking processes, and systemic inhibition may lead to broad-spectrum cytotoxicity (16). Therefore, feasibility assessment must intergrate not only antiviral potency but also tissue distribution, physiological redundancy, and the differential dependency of infected versus uninfected cells on the target.

Toxicity risk is an unavoidable core issue in host-directed therapy, as many host factors involved in viral replication also participate in fundamental cellular processes. For instance, overactivation PKR, a broad−spectrum antiviral effector, can trigger widespread and uncontrolled apoptosis in the respiratory epithelium, causing collateral tissue damage (97). Similarly, long−term inhibition of IDO1, though potentially antiviral, may disrupt immune tolerance and impair neurological function (98). These examples underscore that both hyperactivation and sustained suppression of host factors can be harmful. Therefore, a rigorous toxicity evaluation system must be established before any candidate target can advance to clinical use. Such a system should monitor both direct cytotoxic effects on host cells and indirect immunopathological consequences, using human-relevant *in vitro* models, longitudinal animal studies, and mechanism-based clinical biomarkers to define a safe therapeutic index.

Despite these challenges, several host−targeting strategies have advanced toward clinical application for HPIV, though most remain at the preclinical stage. DAS181 (Fludase), a recombinant sialidase that cleaves sialic acid receptors on the epithelial cell surface, has completed Phase III clinical trails for HPIV infection. It has demonstrated favroable safety and efficacy, particularly in immunocompromised patients, by broadly blocking attachment of multiple respiratory viruses. However, concerns remain regarding its long-term impact on host epithelial barrier function, necessitating continued post-marketing surveillance. Additionally, TMPRSS2 inhibitors such as camostat mesylate, which have accumulated extensive clinical experience in COVID-19 treatment, represent a promising class of repurposed drugs whose potential anti-HPIV activity warrants further exploration (63). These examples illustrate that while clinical translation is progressing, each candidate requires ongoing evaluation of long-term safety and tissue-specific effects.

In conclusion, successful host-targeted therapies for HPIV require a delicate balance among feasible target selection, rigorous toxicity evaluation, and clinical translation progress. While DAS181 has demonstrated favorable safety in immunocompromised patients, long-term epithelial effects warrant follow-up. Ultimately, only through integrated assessment of these three pillars can host-directed strategies become a viable clinical reality for HPIV.

## 5 Perspective and conclusion

Over the past decades, our understanding of virus-host interactions has undergone a profound paradigm shift, moving from a strictly virus-centric perspective toward a deeper appreciation of the complex cellular landscape exploited by viruses. The application of functional genomics and proteomics has greatly expanded our knowledge of host factors involved in HPIV replication, reshaping the view of the infected cell from a passive vessel into an active niche where pro-viral and antiviral dependencies coexist. Looking ahead to the next decade, the field faces several challenges and opportunities.

5.1 Physiological Relevance: Current host factor screening relies excessively on immortalized cell lines, yet transformed cells differ markedly from primary respiratory epithelial cells in metabolism, morphology, and immune thresholds. Therefore, candidate factors must be validated in physiologically relevant models such as human respiratory organoids and air-liquid interface (ALI) cultures.

5.2 Spatiotemporal Dimension: Existing screens provide only static snapshots, but HPIV infection is a highly dynamic chronological process-the host factors required for viral attachment differ from those needed for genome replication or immune evasion. Future studies must integrate temporal multi-omics (transcriptome, proteome, and metabolome) to map the real-time flux of the virus–host interactome, thereby pinpointing transient therapeutic vulnerabilities. At the same time, at the spatial level, viral IBs are not solid aggregates but dynamic membraneless organelles formed via liquid-liquid phase separation. These condensates concentrate the viral replication machinery while selectively recruiting host metabolic enzymes such as PI4KB to build specialized lipid microenvironments (16). This insight points to a novel therapeutic avenue: developing small molecules that rigidify these structures or disrupt the multivalent IDR interactions that sustain their fluidity (99).

In conclusion, the systematic mapping of HPIVs and host interactome holds the key to the next generation of broad-spectrum, host-directed antiviral therapies, ultimately promising to alleviate the global health burden of HPIV-associated respiratory infectious disease.
